# Nanoremediation of arsenic from contaminated water by new generation graphene-based nanomaterials: a comprehensive review

**DOI:** 10.1039/d5ra08599c

**Published:** 2026-02-20

**Authors:** Muhammad Shahbaz Akhtar, Muhammad Atif Irshad, Azhar Hussain, Maria Iqbal, Laiba Ishtiaq, Mohamed Abdel Rafea, Yoshitaka Nakashima, Sami A. Al-Hussain, Ali Irfan, Magdi E. A. Zaki

**Affiliations:** a Department of Environmental Sciences, Forman Christian College (A Chartered University) Lahore Pakistan shahbazakhtar@fccollege.edu.pk laibalaiba065@gmail.com; b Department of Environmental Sciences, The University of Lahore 54000 Pakistan atif.irshad91@yahoo.com azhar1963hussain1993@gmail.com mariakhaan1233@gmail.com; c Department of Physics, College of Science, Imam Mohammad Ibn Saud Islamic University (IMSIU) Riyadh 11623 Saudi Arabia MAKonsow@imamu.edu.sa; d Graduate School of Environmental, Life, Natural Science and Technology, Okayama University Japan nakayosi@okayama-u.ac.jp; e Department of Chemistry, College of Science, Imam Mohammad Ibn Saud Islamic University (IMSIU) Riyadh 11623 Saudi Arabia sahussain@imamu.edu.sa mezaki@imamu.edu.sa; f The Green Institute of Chemical Biomedical and Environmental Sciences (GICBES) Lahore 54000 Pakistan draliirfan.ceo@gicbes.com raialiirfan@gmail.com

## Abstract

Water contamination by metals and metalloids, particularly arsenic (As), is a serious global issue and a pressing challenge for countries with limited water resources. Since As is derived both from natural sources and human activities, including industrial effluents, agricultural runoff, and domestic sewage water discharges, it poses a severe threat to biodiversity, ecosystems, and human health due to its toxicity, carcinogenicity, and mutagenicity, even at trace levels. Traditional remediation methods for As removal from water media, including coagulation, reverse osmosis, and adsorption, are increasingly popular due to low cost and higher removal efficiency. However, recent advances in adsorption research have focused on the development of nanostructured materials with superior physicochemical properties and higher removal efficiencies compared to conventional treatment methods. The high porosity, low density, mechanical strength, and exceptional electrochemical properties of graphene (G)-based nanomaterials distinguish them from other metallic and other polymeric nanomaterials. These new generation GO-based nanomaterials, such as ultra-thin layers of graphene atoms, 2D materials, and nanofibrous sheets, are also efficient remedies for HMs, particularly As, from wastewater. The efficiency and stability of arsenic removal are further improved by nanocomposites, such as GO-polymer hybrids, GO-chitosan, GO-ZnO, GO-cellulose, Fe-functionalized GO, and reduced GO. For environmental remediation, these advanced nanohybrids offer sustainable, high-performance solutions. The present review synthesizes insights from nearly 200 research papers and review articles indexed in Web of Science, Scopus, and Google Scholar, focusing on As removal from wastewater utilizing graphene-based nanomaterials. It also highlights the sources and toxicity of As, limitations of traditional treatment methods, and the enhanced adsorption capabilities of graphene-derived materials and their composites. Overall, this review provides a concise and integrated perspective on current advancements, existing challenges, and future directions for next-generation graphene-based membrane technologies for effective As remediation.

## Abstract 2

Water contamination by metals and metalloids, particularly arsenic (As), is a serious global issue and a pressing challenge for countries with limited water resources.

## Graphical abstract

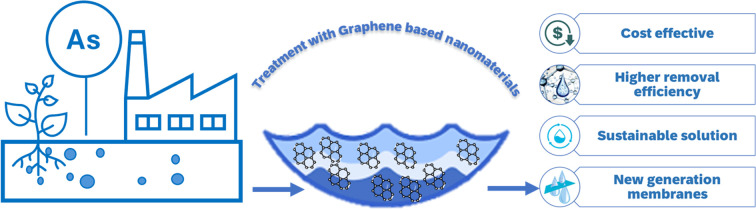

## 1. Introduction

Conservation and protection of water is extremely important for sustainable growth of life on earth. Day in and day out, depletion and decline of global freshwater resources is resulting in water scarcity. Contamination of freshwater and soil with inorganics (metals and metalloids) and organics (dyes, persistent organic pollutants (POPs), polychlorinated biphenyls (PCBs), and polycyclic aromatic hydrocarbons (PAHs), from domestic effluents and industrial discharges is a serious global issue.^1–3^

In earth's crust, minerals, soils, groundwater, and living organisms, arsenic (As) is one of the most toxic metals and metalloids. Additionally, As is used in wood preservation; fertilizer and pesticide manufacturing; and metallurgical mining, glass, and semiconductor formations.^4^ It is estimated that As is released into the environment between 52 000 and 1 12 000 tons annually, and it is regarded as a class-1 contaminant and a hazardous pollutant due to its high persistence and low degradation. The WHO estimated an annual global exposure of As (≥10 µg L^−1^ in water) to more than 180 million people around the globe. Arsenic toxicity and bioaccumulation capacity depend on As chemical species and the metabolically mediated biomagnification process.^5,6^ Arsenic species can exist in four oxidation states (−3, 0, +3, +5), and unfortunately both organic and inorganic forms are toxic to humans. Pentavalent As(v) is the most prevalent form in an oxidized environment (high Eh value), and its distribution has been related to pH. Under natural environmental conditions (pH = 4–8), H_2_AsO_4_, and HAsO_4_^2−^ are anionic forms of As(v) along with other metal cations that are naturally occurring in waters.^7,8^ Once released into the environment, these contaminants are a serious concern for the environment and pose health issues *via* food chains and biomagnification processes.^9,10^

Arsenic removal from water bodies is achieved by a variety of procedures, including filtration/separation, coagulation/flocculation, electrocoagulation, ion exchange, and sorption/adsorption.^11,12^ Among these removal techniques, sorption is regarded as the most cost-effective, simple, and easily implementable technique. Due to the synthesis of nanostructured sorbents and nanomaterials in recent years, this approach has gained popularity in the removal of toxic substances from water and soil.^13–15^ Due to their high specific surface area, distinct physicochemical features, and increased availability of functional groups, these nanomaterials have a unique sorption potential. Among the plethora of nanomaterials, new-generation graphene (G)-based materials are key players and highly effective for decontamination of pollutants from soils and waters. Unique physicochemical characteristics of graphene and graphene-based materials, such as high specific surface area, more reactive sites and functional groups, better sorption efficacy, high porosity, low density, mobility, chemical suitability, ionic strength, and electrochemical performance, make these G-based materials super sorbents. New generation super sorbents have superior surface chemistry, structure, properties, and functional groups for higher reactivity and their ability to selectively adsorb specific molecules. This makes them highly efficient in applications such as water purification, cleanup of oil spills, and chemical processing, where traditional sorbents often fall short in performance and versatility.

In this review, key themes such as arsenic occurrence, toxicity, and limitations of current treatment methods of removal from wastewater using graphene-based nanomaterials are discussed, providing a detailed assessment of the adsorption performance of carbon-based nanomaterials and graphene-derived nanocomposites. The criterion for new-generation graphene includes nanomembranes that are more effective than traditional GO-based nanocomposites. Graphene, as the first known crystal of carbon, has better efficiency than typical nanomaterials, particularly carbon-based nanomaterials. In addition to the extensive literature on As adsorption utilizing ordinary graphene and its derivatives, this review provides some insights for improving and advancing work using nano graphene membranes to make it more compatible, usable, and effective. As(iii) and As(v) removal mechanisms are clarified in the review of recent advances in graphene-based fabricated membranes. This article presents a comprehensive overview of current advances, research gaps, and potential opportunities for developing efficient and sustainable graphene-based technologies for As remediation by combining these diverse aspects into a single framework.

## 2. Carbon-based nanomaterials and nanocomposites

**Fig. 1:**
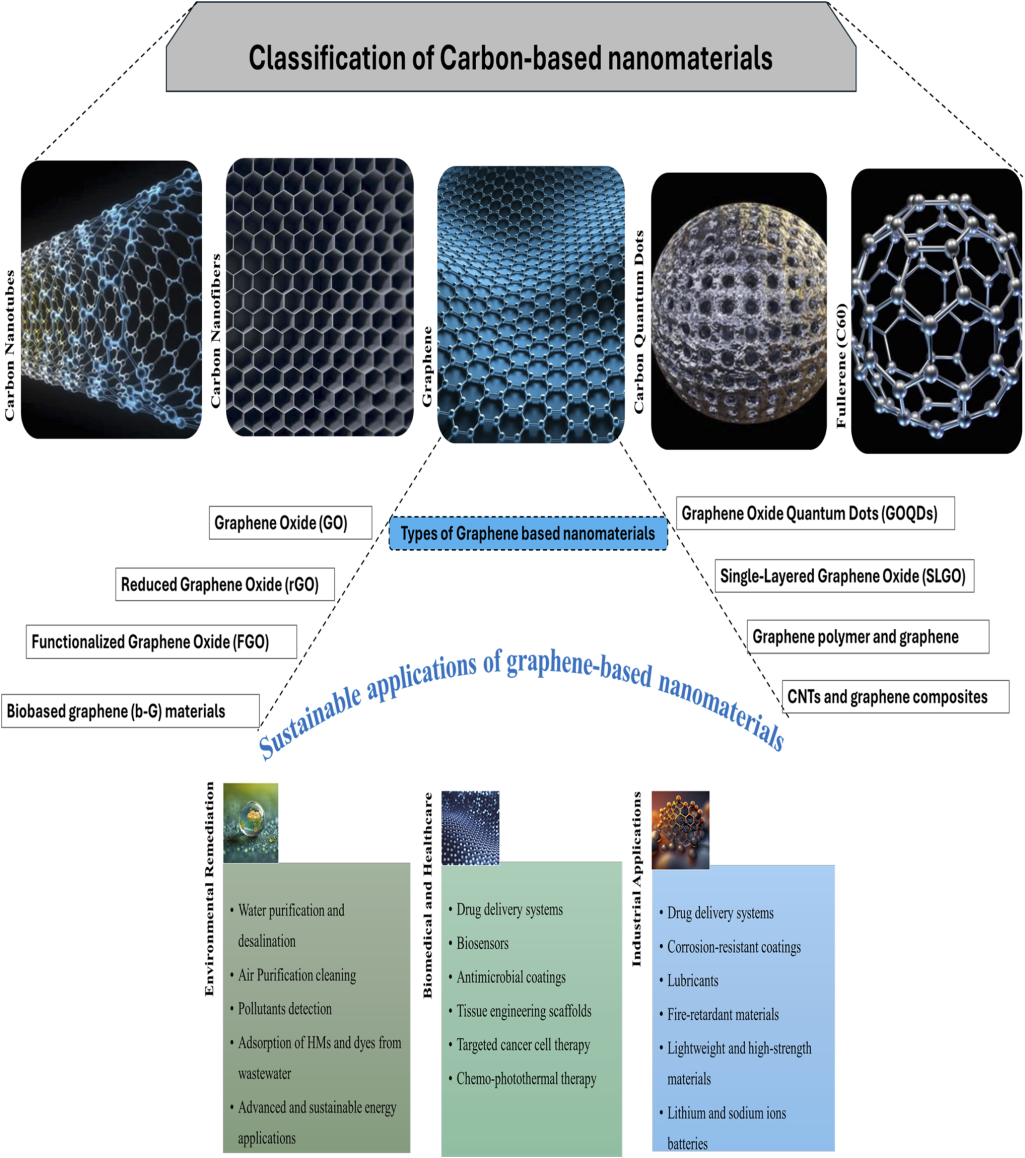
Classification of carbon-based nanomaterials classified with major types with special focus of graphene-based nanomaterials, their types and sustainable applications.

Carbon-based nanomaterials (CBNM) are a type of nanoscale material with outstanding physical, chemical, and mechanical capabilities, making them extremely important in a wide range of research and industrial applications. In addition to zero-dimensional fullerenes and carbon quantum dots, these materials include one-dimensional (1D) carbon nanotubes and nanofibers, two-dimensional (2D) graphene sheets, and three-dimensional (3D) nanodiamonds and carbon nano-onions.^16,17^ They are widely used in electronics, energy storage, catalysis, environmental remediation, biosensing, and biomedical engineering due to their high surface area, exceptional electrical and thermal conductivity, superior mechanical strength, and chemical tunability. Following Fig. 1 outlined the major classification of carbon-based nanomaterials along with the focus on graphene and its types with sustainable applications.

### 2.1 Types of graphene oxide nanomaterials

Graphene represents the preliminary discovery of a two-dimensional crystal by humankind. Comprising a singular graphite sheet, its distinctive characteristics are revolutionizing material research. Graphene nanoparticles present significant appeal owing to their flat structure, lightweight nature, high aspect ratio, excellent electrical conductivity, affordability, and robust mechanical properties. Graphene and its derivatives, including graphene oxide and reduced graphene oxide, along with various types of graphene, are outlined as follows.

#### 2.1.1 Graphene oxide (GO)

Nonstoichiometric GO with differential compositions is synthesized depending on the synthesis environment. These approaches based on chemical exfoliation and oxidation of graphitic flakes include the Brodie protocol (oxidizing mixture of KClO_4_ + fuming HNO_3_ synthesizes GO with graphitizable-C of graphite).^18^ During synthesis of GO, the exfoliation process is carried out using different solvents *via* an ultrasonic approach followed by sonication.^19,20^ More recently, GO exfoliation and reduction can be carried out by different methods, such as rapid heating (thermal), mechanical method (stirring), chemical, plasma, and microwave methods.^21^ The synthesis of GO by Hummer's approach displays disrupted conjugated graphene planes and modified surfaces containing different functional groups such as carboxyl (COOH, carbonyl(C=O), hydroxyl (OH), and epoxide (C–O–C). After graphitic chemical oxidation, graphene layers are decorated with OH and C–O–C groups on basal plane functional groups and COOH as terminal end functional groups. The characteristics of GO, like hydrophilicity and negative charged density, can be attributed to the decoration of O-containing functional groups.^22^ Functionalized single layers of GO with C–O–C, OH, and COOH functional groups are characterized with higher (0.7–1.2 nm) thickness than the pristine graphene thickness (0.335 nm).^23^ Upon oxidation of bulk materials, synthesized GO is a solid product that still has a graphitic layering structure but with wider and erratic spaces and has a C : O ratio between 2.1–2.9. The oxidation-based GO sheet characterization and separation can be accomplished *via* fractionation techniques that can be ascribed to polydispersion of distributed oxygen on GO. Different techniques are in practice to synthesize different forms of GO. Synthesis of GO by modified Hummer's protocol *via* chemical exfoliation and oxidation approach.

#### 2.1.2 Reduced graphene oxide (rGO)

Reduced graphene oxide (rGO), a derivative of GO, is characterized by a low C : O ratio compared to GO and can be synthesized successfully by a sequential reduction process *via* chemical, thermal/pyrolysis, and other processes. The rGO has a close resemblance to pristine graphene. Major synthesis methods include thermal annealing, microwave-assisted laser irradiation and light-induced reduction hydrothermal methods, and chemical reduction by reducing agents, such as ascorbic acid (C_6_H_8_O_6_), hydrohalic acids (HX where X= halogens), sodium borohydrate (NaBH_4_), aluminum hydride (AlH_3_), hydrazine hydrate (N_2_H_4_), and thiourea (CH_4_N_2_S).^24,25^ Among reducing agents, N_2_H_4_ is the most used as oxygen molecules scavenger and N_2_ and H_2_O molecules are formed during reaction processes.

#### 2.1.3 Functionalized graphene oxide (FGO)

Graphene-based nanomaterials can be functionalized *via* covalent and noncovalent approaches by modifying their surface chemistry, such as surface functionalization due to rehybridization of one or more sp^2^ C atoms into Sp^3^ C atoms in carbon systems, and by enhancing specificity and solubility.^26,27^ Graphene exterior surfaces modified by strong oxidants (KMnO_4,_ H_2_SO_4_ and HNO_3_) due to the addition of –OH and –COOH are ascribed to covalent functionalization. For example, possible hydrogenation of graphene by atomic H on SiC-supported G and Ir (111), and G-derivates with fluorine (F) due to more affinity of F to C than H, and fabrication of G-surfaces with sulfonate (–SO_3_, –SO_3_H) and amino (–NH_2_) groups. Nondestructive changes in characteristics of graphene-based materials without changes in structural arrangement can occur by non-covalent functionalization *via* van der Waals, hydrophobic, and electrostatic interactions. Examples of non-covalent functionalization of G-based materials include robust affinity of pyrene butyric acid C_20_H_16_O_2_*via* π-stacking, linking molecules with a tail (aromatic linkers) or end (responsive linkers). Likewise, G-decking with metallic nanoparticles (Ru, Au, Pt, Ni, Ag *etc.*) *via* thermal evaporation, reduction (*in situ*) process, electrical deposition and photochemical processes.

#### 2.1.4 Biobased graphene (b-G) materials

More recently, green chemistry approaches/methods are becoming more popular because these are ecofriendly and sustainable. Plants and animal-based biomass and biomaterials are available in bulk and renewable in nature. These biomass/biomaterials are precursor materials for carbon sources, and their reuse will minimize waste. Biomass waste/agrowastes are being used for the synthesis of G-based materials, which have advantages in terms of sufficient availability and their carbon-rich structure. Biomass-derived bio-based graphene (bG) can be synthesized by pyrolysis/thermal approach and carbon growth. Removal of volatile matter contents from polymeric chains of biomass *via* thermal processes can enhance carbon content. Like the graphitic route, bio-based graphene oxide (bGO) and bio-based reduced graphene oxide (brGO) can be synthesized using the non-graphitic route. Nonetheless, bG-based materials synthesized from biomass or biomaterials display some similarity with materials synthesized from a non-graphitic route, however, bG-based materials display slight differences in structure and characteristics.

#### 2.1.5 Single layered graphene oxide

Graphite is a carbon material crystalline in nature with fixed planes and similar layers with covalent and metallic intraplanar bonding greater than interplanar van der Waals forces. Graphite acts as a precursor of graphene (G), as a stack of graphene is known as graphite. Graphite layers have regular hexagons with fixed carbon atoms with interlayer spacing of 3.35 Å. The carbon atoms are detached (carbon-to-carbon distance) by 1.42 Å in a graphitic hexagonal array. Graphite is highly anisotropic in structure due to differential interplanar and intraplanar properties.^28^ Due to the reactivity of graphite with different chemicals and reagents, different intercalation compounds can be synthesized *via* surface substitution and modifications.^29^ High sorption capacities and unique surface chemistry of graphite intercalation compounds might offer promising solutions for decontamination of pollutants from water and soils. Graphene is sp^2^ hybridized single monomolecular layer (2-D sheets) characterized with a honeycomb lattice structure decorated with a high number of oxygen-containing functional entities orderly arranged on both sides of 3-D graphitic sheets.^30^ This 2-D material is structured with benzene rings along with stuffed carbon atoms and striped of hydrogen atoms. Pristine graphene is an original, pure, and unoxidized form of graphene having high carrier mobility. Synthesis of graphene is possible by different protocols/methods such as cleavage (micromechanical), arc discharge, CVD (chemical vapor deposition), growth (epitaxial) on SiC (silicon carbide), electrochemical approach, C-nanotube unzipping, chemical reduction of GO, total organic synthesis, and plasma discharge induced etching of graphite.^31^ Nevertheless, graphene synthesized by these methods might not be practical for environmental applications because the synthesis of graphene should be cost-effective, efficient, and on a large scale. Furthermore, pristine graphene displays less dispersion in water for removal of pollutants due to its hydrophobic nature. However, the recent study of pristine graphene in removing arsenic from wastewater is found promising due to its strong adsorption character and high surface area. It happens since pristine graphene in both vacuum and aqueous conditions can efficiently fix arsenic through different adsorption sites, including edges and bridge positions on its surface, achieving strong interaction energies.^32^

Among the family of carbon nanomaterials, graphene (G) gained much interest in the current arena after its discovery by Andre Geim and Konstantin Novoselov in 2004, who synthesized graphene by stripping off the graphite using adhesive tape *via* the mechanical exfoliation approach (Novoselov *et al.*, 2004). Exfoliation of graphitic layers *via* a nanomanipulation approach to extract and characterize graphene was started much earlier by the pioneer work.^33^ Two major routes are generally adopted to synthesize graphene.^34^ In the top-down approach, graphitic layers are exfoliated, whereas in the bottom-up approach, the growth of small (molecular-sized) C-precursors is ensured to synthesize nanomaterials.^35–38^ To overcome the solubility issue of pristine graphene, modification and fabrication in graphene is generally done by introducing different surface functional groups *via* chemical modification and functionalization (covalent or noncovalent). Functionalized graphene family members include different carbon nanomaterials such as graphene oxide (GO), reduced graphene oxide (rGO), functionalized G-derivatives, and G-nanocomposites. These carbon-based nanomaterials and nanocomposites have different sizes, shapes, orientations, surface chemistry, morphological and physicochemical characteristics.^39^

#### 2.1.6 Graphene quantum dots (GOQDs)

Graphene oxide quantum dots (GOQDs) are ultra-small, zero-dimensional carbon nanostructures derived from graphene oxide, with lateral dimensions typically below 100 nm and oxygen-containing functional groups. Their structure combines the robust, sp^2^-hybridized carbon framework of graphene with the enhanced solubility and chemical reactivity imparted by surface oxygen groups, resulting in excellent fluorescence and tunable band gaps *via* quantum confinement and edge effects in aqueous environments.^40^ These characteristics make GOQDs very appealing for a variety of biomedical applications: they can activate the Wnt/β-catenin signaling pathway to promote osteogenic differentiation of human stem cells,^41,42^ and when combined with hyaluronic acid, they function as dual-function nanocarriers that have the ability to deliver drugs both therapeutically and specifically, such as increasing the effectiveness of metformin in metabolic inflammation models.^43^ All things considered, GOQDs' distinct blend of bioactivity, tunable optical behavior, and adaptable functionalization make them an extremely attractive platform for nanotherapeutics, regenerative medicine, and next generation bioimaging.

#### 2.1.7 Graphene based composites

Graphene-based nanomaterials and nanocomposites could be promising and potential super sorbents for environmental contaminants because of their unique physicochemical properties (higher surface area and delocalized π-electrons) and superior surface chemistry. Nevertheless, strong interplanar interactions can result in restacking G-nanolayers into graphitic layers, due to which binding affinity of GO for different compounds/substances can occur due to sturdy electrostatic repulsion and less detachment of G-based materials. Functionalization of G *via* covalent or non-covalent interactions with different nanomaterials and dissimilar molecules can overcome these limitations. These synthesized nanocomposites are multicomponent materials where one phase is dispersed into another phase in the nanometric range.

#### 2.1.8 Graphene polymer and graphene carbon nanotubes composites

The G-polymer composites and G-carbon nanotube composites can be synthesized in addition to G-based nanocomposites. Carbon nanotubes (CNTs) are carbon allotropes with rolled up G-sheets having long, thin, and cylindrical shapes. Improvement in compatibility and distribution of CNs and GNs can be done due to surface reactive groups modified with phenol-formaldehyde resin that will result in possible synthesis of different nanocomposites such as glassy C and C/C composites. Likewise different G-polymer composites such as polyaniline, polystyrene, polyvinyl alcohol, polycarbonate, and Nafion are reported in pertinent literature.^43,44^

## 3. Arsenic occurrence and exposure, environmental contamination and health impacts

**Fig. 2:**
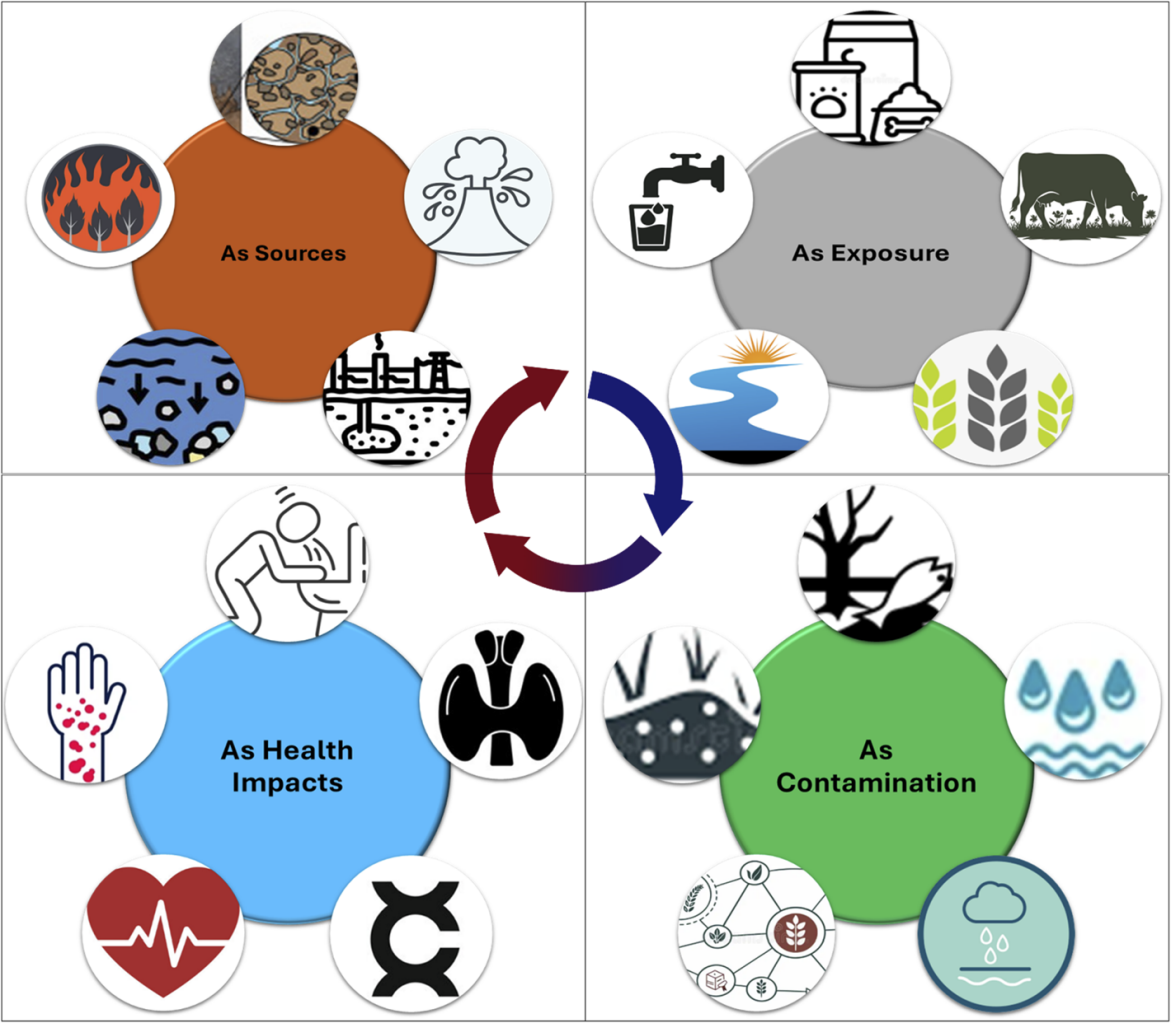
Arsenic in nature: from sources to direct health impacts to human beings, exposure and contamination to environment.

Arsenic (As) is found in varying concentrations in water bodies throughout the globe. The higher level of As exposure may cause instant and long-standing health problems, comprising heart disease, cancer, childhood cancer, miscarriage, diarrhea, vomiting, diabetes, and even loss of life. The World Health Organization (WHO) recommends a maximum allowable concentration (MAC) of As in drinking water at 10 µg L^−1^ to ensure safe drinking water and protect public health. After being released into the surroundings, it becomes part of our biogeochemical cycle and thus unable to be degraded. It suffers compound chemical speciation in aquatic environments, causing a variety of organic and inorganic arsenic species. The organic forms of As include methylarsonate (MMA), dimethylarsinate (DMA), tetramethylarsine (TMA), trimethylarsine oxide (TMAO), arsenocholine (AsC), arsenobetaine (AsB), thiolated arsenic, and arsenosugars (As-Sug) *etc*, while inorganic forms of As consists of arsenite As(iii) and arsenate As(v).^45^ However, in water, As exists in various redox states, mostly as inorganic types such as As(iii) and As(v). It is noticed that organic species are more common when there is anthropogenic contamination. Moreover, As(iii) is extensively renowned for its greater toxicity compared to As(v). Through the water and diet chain, when As mixes into aquatic organisms, they start to accumulate, retain, and alter different forms of arsenic within their structures.^46^ Arsenic participation in nature, its absorption and assimilation, biotransformation, and ultimately harm the ecosystem and human health. The main bioaccumulation paradigm is illustrated in Fig. 2.

After As is captivated by an organism, it could undertake biotransformation of a more toxic form of As to convert into a less toxic As in an aquatic environment. The inorganic arsenic (iAs) is more dangerous than organoarsenic in terms of toxicity and is considered a verified pollutant for humans. Also, arsenite As(iii) is classically more toxic than arsenate As(v), while monomethylarsonous acid (MMAAIII) and dimethylarsinous acid (DMAAIII) show greater toxicity than their respective parent compounds.^46^ Although there are some exceptions in aquatic systems, inorganic arsenic (iAs) is typically more harmful than organo-arsenic compounds. Species and arsenic form affect toxicity: freshwater phytoplankton is more sensitive to arsenate As(v) than sea phytoplankton (Dunaliella sp. and *Polyphysa peniculus*) to arsenite As(iii). This variation emphasizes the necessity of toxicity evaluations that are species- and environment-specific rather than generic.

## 4. Arsenic removal techniques from wastewater

**Fig. 3:**
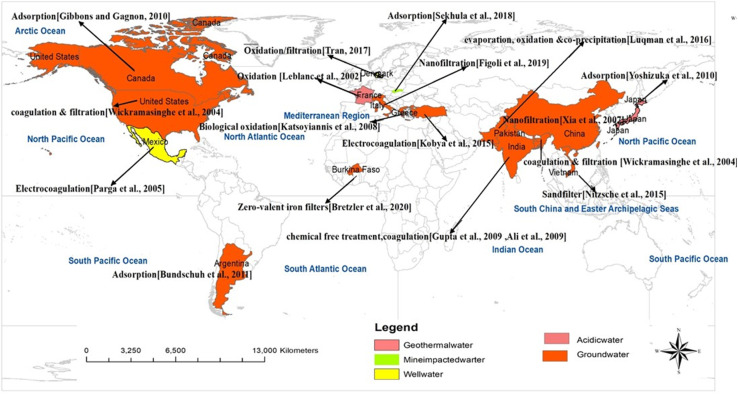
Global distribution of As removal from wastewater utilizing the different advanced and efficient techniques.

Work on graphene discovery started much earlier than the graphene extraction and characterization by Novoselov *et al.* (2004), such as the nanomanipulation approach focusing on the exploitation of a few graphitic layers.^33^ Two approaches are generally adopted in graphene synthesis, *i.e.*, a top-down and a bottom-up approach.^47^ Graphite or graphitic derivatives are separated or exfoliated to synthesize graphene in a top-down approach, while graphene is synthesized by the growth of small molecular precursors of carbon in a bottom-up approach. Both approaches have their own merits and demerits, which were documented in pertinent literature.^35–38,47,48^ Globally, a variety of technologies are applied for the removal of As from water bodies, which are given in Fig. 3. The removal of As from water sources can be undertaken by various techniques such as electrocoagulation, membrane filtration, chemical precipitation, ion-exchange, phytoremediation. and adsorption.

### 4.1 Electrocoagulation

By using the technique of electrocoagulation (EC), As can be rejected by using ferric chloride, ferric sulfate salts, and alum, *etc.*, being common and famous materials for effective As removal from water. However, on a weight basis, the iron salts are found to be more effective as compared to alum over a wider pH range in eradicating As than alum. By using EC technique, the fully automated high-tech EC reactor can be used on a large-scale for As removal from water sources.^49^ The fully automated EC reactor can remove As(iii) and As(v) from contaminated water with an impressive efficiency range of 93% and 99.9%.^50^

### 4.2 Membrane filtration

The technique of membrane technology offers an excellent option for As removal from groundwater. It can prevent bacteria from piercing the membrane and ultimately ejecting As from polluted water. The latest membrane-based processes for the capture of As and a variety of other dissolved particles from water include ultrafiltration (UF), reverse osmosis (RO), microfiltration (MF), and nanofiltration (NF). Among various membrane techniques, the RO and NF techniques are found to be very effective for As(iii) and As(v) removal from polluted water bodies in different environments. Moreover, the efficiency of As removal greatly depends upon the pore size of the membrane filters and the type of membrane technique used for water purification.^51^

### 4.3 Ion exchange technique

The removal of As by a physicochemical action during which electrostatically seized ions on the solid phase are exchanged by ions having a similar charge present in the solution. This technique is suitable for removing various chemicals from wastewater.^52^ However, the drawback of ion exchange technique is that it releases reagents in water during regeneration of resin which is hazardous chemical for living organisms.

### 4.4 Phytoremediation

Phytoremediation is a technique of removing various toxic contaminations from water sources, including As, by using a variety of plants. For instance, the Chinese brake fern, named *Pteris vittata*, showed a great aptitude to absorb As from water bodies. During water purification, the phytoremediation is supplemented alongside the techniques of phytovolatilization, phytostabilization, and phyto extraction, respectively. During phytoremediation, the desired impurities of contaminated water are successfully absorbed by the plant roots and then transported to the outer ground parts of the plants and then are safely removed along with the crops grown in the polluted water environments. Nowadays, phytoremediation joins hands with green nanotechnology called nanophytoremediation (NP) to clean up polluted environments in a sustainable way. The phytostabilization used for water purification drastically lowers the chances of As transfer in food chains since the vegetative biomass above ground is not polluted with As, which is its principal benefit. So, using NP in union with a variety of plants, the polluted water sources can be cleaned up from As adulterations accordingly.^53^ It is noticed that phytoremediation taken up with nanomaterials has been found very effective in water purification systems even though it is time-consuming.^54^

### 4.5 Adsorption technique

The adsorption technique is found to be very effective in removing As due to its ease of operation, sludge-free nature, high efficiency, and low cost as compared to other techniques. Adsorption of As in water depends on the van der Waals separation process and the electrostatic forces present between molecules of the solution.^55^ As(iii) and As(v) can be effectively removed by adsorption with an impressive efficiency of more than 95% As removal. However, this adsorption success is associated with factors like initial As concentration, amount of adsorbent, surface temperature, pH, presence of other chemical contaminations, and exposure time.^56^ It is verified that the engineered nanoparticles can display adsorption levels for As(iii) beyond 100 mg g^−1^ (Liu.*et al.*, 2019). Moreover, the extreme adsorption capacity of engineered adsorbents is greater than that of single adsorbents for As removal from water bodies.

## 5. Adsorption of As using graphene-based nanomaterials

Nowadays, As removal from water can be efficiently done using activated graphene-based adsorbent sources like carbon and biochar. Graphene oxide-based nanomaterials provide much greater adsorption than the other nanomaterials and can efficiently remove heavy metal ions such as As from wastewater on a large-scale using graphene-based adsorption methods.^57^ Due to the large surface areas of graphene-based materials such as reduced graphene oxide, strong mechanical strength, and plenty of oxygen-containing useful groups, it can be adsorbed As ions on its surfaces.^58,59^ In one of the studies, adsorbing both As(iii) and As(v) from contaminated soil by use of composite material of graphene oxide combined with copper ferrite (GO/CuFe_2_O_4_) was found to be very effective. This research verified that the GO/CuFe_2_O_4_ has an adsorption capacity of 51.64 mg g^−1^ for As(iii) while for As(v) the adsorption reached to the level of 124.68 mg g^−1^. Because of its high adsorption values of 306 mg g^−1^ for As(iii) and 431 mg g^−1^ for As(v), respectively, the composite material of graphene oxide functionalized with iron nanoparticles (FeNP@GO) has been discovered to be one of the most effective materials for the removal of arsenic. Through complexation and electrostatic attraction, As(iii) and As(v) are bound to the iron nanoparticles on the GO surface, causing this adsorption.^60^ Additionally, As(v) can be effectively removed and absorbed by magnetite-reduced graphene oxide (Mag-PRGO). With an absorption capacity of 131.9 mg g^−1^ for As(v), this mag-PRGO is one type of graphene-based adsorbent that works with magnetite (FeO_4_) particles. Magnetite-reduced graphene oxide shows magnetic properties, which permit easy split-up of the adsorbent after arsenic removal from the water or soil. The use of quantum dots with graphene oxide is also an effective medium for As removal from the soil. In one such study the fabricated material joined with iron oxyhydroxide quantum dots onto graphene oxide (α-FeOOH QDs@GO) yielded an adsorption capacity of 147.38 mg g^−1^ for As(iii) and 69.03 mg g^−1^ for As(v) respectively. It happens due to the facts that the α-FeOOH nanoparticles aid to raise both the porosity and absorbing surface area in raising the higher arsenic uptake from the contaminated soil. It is noticed that the arsenic metal can also be effectively removed from water by use of functionalized graphene materials, for instance graphene oxide (GO) composites. The surfaces of this functionalized graphene material offer greater adsorption capacity, making it highly active for seizing both arsenic(iii) and arsenic(v) impurities from water. Moreover, these amendments increase the surface area and chemical interactions between arsenic ions and GO. Due to these characteristics, functionalized graphene provides a favorable and proficient way out for large-scale environmental applications in handling arsenic-contaminated water.^39^

A recent study of optimized GO in eradicating arsenic from water verified that different oxidation levels impact the adsorption capacity of GO accordingly. That is, as the oxidation of GO increases, its capacity to arrest arsenic, predominantly As(iii), also increases. It was noticed that enhanced GO can adsorb up to 288 mg g^−1^ of As a remarkable improvement from former values of As(iii). Hence, optimized GO behaves as an active material, proposing an accessible answer for removing arsenic from contaminated sources of wastewater.

The polyethyleneimine-functionalized graphene oxide (PEI-GO) has also been used for the elimination of arsenic from contaminated water sources. The PEI-GO functionalization increases GO's adsorption capacity by increasing its contact with arsenic impurities, primarily arsenic(iii) and arsenic(v), respectively. This PEI-GO amendment presents additional functional groups that permit tougher electrostatic attraction, boosting the material's efficiency in interacting with arsenic ions. This research reveals that PEI-GO is an effective material to be used in water treatment applications, offering a promising approach for the selective removal of arsenic from contaminated water sources. Arsenic concentrations in water bodies around the world are summarized in Table 1, with Nicaragua having the highest level in drinking water (1320 µg L^−1^). These concerning levels in groundwater, wastewater, marine life, and drinking water emphasize how urgently efficient removal techniques are needed. Chandra *et al.*^61^ effectively extracted arsenite As(iii) and arsenate As(v) from water using modified magnetic graphene oxide (GO).

**Table 1:** Synthesis, characterization and experimental setup of different graphene-based nanomaterials for the removal of As from wastewater

S. No.	Type of GO-based nanomaterials	Synthesis method	Type of treated water	Experimental set-up	Kinetic/isothermal modelling	Results and outcome	References
1	Magnetite–Graphene oxide (MGO)/Magnetite–rGO (MRGO)	Magnetite nanoparticles decorated onto GO or rGO (co-precipitation or *in situ* growth on GO sheets)	Synthetic As(iii)/As(v) solutions; spiked groundwater	Batch adsorption; column tests; magnetic separation for recovery	Pseudo-second order/Langmuir, Freundlich, Redlich–Peterson	Magnetite-graphene hybrids (∼10 nm) showed high As(iii) and As(v) adsorption achieved up to 99.9% removal efficiency	61
2	Magnetic graphene oxide (recent pilot/cost-effective syntheses)	Cost-effective synthesis variants and scaled-up MGO routes	Ground water, wastewater	Batch study, column tests, magnetic recovery & lifecycle assessment	Pseudo-second order/Langmuir	MGO exhibited chemisorption and intra-particular diffusion, five times higher As adsorption capacity than GO and can persist after five reuse cycles	62
3	GO/CNT/Fe_3_O_4_ ternary composite	Co-precipitation of Fe_3_O_4_ onto GO/CNT scaffold	Wastewater/synthetic arsenic solutions	Batch adsorption, magnetic separation	Pseudo-second order/Langmuir, Freundlich	GO/CNT/Fe_3_O_4_ highly selective for competing ions, followed Langmuir isotherm (*R*_2_ = 0.998), and the maximum As(iii) adsorption capacity (*q*_max_ 128.5 mg g^−1^) with approximately 99.18% removal efficiency	63
4	GO–goethite–chitosan composite	GO composit with goethite and crosslinked chitosan beads	Synthetic arsenic solutions	Batch adsorption, bead immobilization, regeneration cycles	Pseudo-second order/Langmuir	GO/goethite-chitosan composite was found to have high As(iii) adsorption (368.38 mg g^−1^ at pH 6, with 98.96% removal efficiency	64
5	Magnetic graphene oxide (MGO, Fe_3_O_4_@GO)	Co-precipitation of Fe_3_O_4_ on GO (magnetic functionalization)	Synthetic As(iii)/As(v), spiked groundwater	Batch + column tests; magnetic separation & reuse	Pseudo-second order; Langmuir/Freundlich	MGO- Fe_3_O_4_ (surface complexation and H-bonding were the major processes, with removal efficiency 98% of As(iii) in 20 minutes	65
6	GO–granular ferric hydroxide (GFH) hybrid	Physical/chemical combination of GO with GFH (mixing/immobilization)	Aqueous As(iii) solutions, drinking water simulations	Batch tests (pH, dose, contact time optimization)	Pseudo-second order, Langmuir, Freundlich	GO/GFH composite enhanced the elimination of As(iii) to 90% at a 2 : 1 ratio (0.2 g L^−1^ GO + 0.1 g L^−1^ GFH)	66
7	UiO-66-NDC/GO (MOF-GO nanocomposite)	Solvothermal synthesis of Zr-MOF grown with GO sheets (MOF/GO composite)	Synthetic As(v) solutions	Batch adsorption, regeneration (acid desorption) and reusability tests	Pseudo-second order/Langmuir	UiO-66-NDC/GO had high As(v) adsorption (*q*_max_ 147.06 mg g^−1^, and was spontaneous and exothermic, and reusable after four rounds of regeneration	67
8	FeOOH-GO hybrids (α-FeOOH@GO)	Ligand-modulated β-and α-FeOOH impregnation on GO	Synthetic As(iii)/As(v) solutions	Batch adsorption; structural variation	Not explicitly mentioned	The highest As(iii) adsorption was observed in 4.6× greater than in other FeOOH materials, and the adsorption was due to the interactions between hydroxyl-group with other FeOOH materials	68
9	Magnetic graphene oxide ion-imprinted polymer (MGO-IIP)	Magnetized GO combined with ion-imprinted polymerization for As(iii)	Synthetic As(iii) solutions	Batch adsorption; selectivity and regeneration tests	Kinetics: Pseudo-second order; selectivity assays	MGO-As(iii)-IIP showed the maximum adsorption capacity of 49.42 mg g^−1^ with a high selectivity of 75% capacity after five reuse cycles	69
10	GO–Fe (iron oxide) hybrids/GO–FeO_*x*_–chitosan composites	GO chemically combined with iron oxides or Fe-modified chitosan beads (co-precipitation/crosslinking)	Synthetic arsenate/arenite solutions; drinking-water relevant matrices	Batch tests and column/column-like bead tests; pH and dose studies	Pseudo-second order/Langmuir, Freundlich	GO–chitosan–iron oxide beads (∼1 mm) rapidly reduced As(iii) to safe levels (<6 h), showed stable performance	70
11	GO–clinoptilolite (Fe-modified clinoptilolite) composites	GO composited with iron-modified zeolite (clinoptilolite)	Synthetic arsenate solutions, simulated waters	Batch adsorption, column experiments	Pseudo-second-order, column test	GOFeZA and GOFeZB had high arsenate adsorption (557.86 and 554.64 µg g^−1^ at 450 µg L^−1^), followed pseudo-second-order kinetics *via* surface complexation, ion exchange, and electrostatic attraction	71
12	Graphene oxide–iron nanohybrid (GFeN)	Sol–gel deposition of Fe/Fe_*x*_O on GO	Synthetic As(iii)/As(v) solutions	Batch adsorption; characterization (BET, TEM)	Pseudo-second order; Langmuir	GFeN nanohybrid showed high As(iii) and As(v) adsorption (306 mg g^−1^and 431 mg g^−1^) with >99% removal in <10 min with GO sheets enhancing FeNPs efficiency and reusability	72
13	Magnetic GO derivatives	Magnetic functionalization of GO	Spiked groundwater/real As contaminated water	Batch + column tests; magnetic separation and regeneration cycles evaluated	Pseudo-second order kinetics and Langmuir/Freundlich isotherms	MGO-IL rapidly removed arsenic (*q*_max_ 160.65 mg g^−1^for As(iii), 104.13 mg g^−1^ for As(v)) in 30 min, showed excellent reusability (>5 cycles) with easy magnetic separation, and ionic liquid modification enhanced adsorption efficiency and speed	45
15	Reduced graphene oxide (rGO) – TiO_2_ or Ti-based composites	rGO combined with TiO_2_*via* hydrothermal/sol–gel routes	Aqueous As(iii)/As(v) solutions; sometimes solar/photocatalytic contexts	Batch adsorption; some studies combine oxidation (As(iii) → As(v)) + adsorption	Pseudo-second order kinetics/Langmuir, Freundlich	rGO–TiO_2_@fibers oxidized As(iii) 2.5× faster than GO-TiO_2_@fibers, with enhanced hydroxyl radical generation from rGO–TiO_2_ preventing electron–hole recombination, showing low Ti leaching and stable performance over multiple cycles for reusable water treatment	73
16	3D magnetic GO hydrogel (MGOH)	Assembly with GO into 3D hydrogel	Synthetic As(iii)/As(v) solutions	Batch adsorption; macroporous hydrogel format	Rapid kinetics	3D MGOH rapidly adsorbed As(iii) and As(v) (25.1 mg g^−1^ and 74.2 mg g^−1^) within 2 min, with oxygenated moieties preserved *via* chemical bubble functionalization	74
17	rGO-supported mesoporous Fe_2_O_3_/TiO_2_ (rGO–Fe_2_O_3_/TiO_2_)	Sol–gel synthesis supporting Fe_2_O_3_/TiO_2_ on rGO	Aqueous As(iii)/As(v) solutions, photocatalytic contexts	Batch adsorption + photocatalytic oxidation setups	Pseudo-second order/Langmuir, Freundlich	Fe_2_O_3_/TiO_2_ (rGO composite rapidly and efficiently adsorbed As ions across a wide pH range with high selectivity, good reusability, durability, and potential for sensing applications	75
18	FeO_*x*_–GO nanocomposite	*In situ* growth of iron oxide on GO (Fe : GO ratios)	Synthetic As(iii)/As(v) solutions	Batch adsorption; characterization by XRD/SEM	Pseudo-second order/Langmuir, Freundlich	FeOxGO-80 nanocomposite showed high As adsorption (147 mg g^−1^ for As(iii), 113 mg g^−1^ for As(v) reduced arsenic levels to <0.02 mg L^−1^	76

GO-based iron and magnetic nanocomposites have received significant attention as next-generation adsorbents for arsenic remediation due to their high surface area, abundant oxygenated functional groups, and strong affinity for arsenic species. MGO and GO–Fe consistently outperforms pristine GO, mainly because of the additional active sites introduced with iron-based functionalization, which significantly enhance chemisorption, surface complexation, and hydrogen bonding with As(iii) and As(v).^61,65,66,76^ Comparatively, iron oxide–decorated GO materials such as Fe_3_O_4_@GO, FeO_*x*_–GO, and FeOOH–GO exhibited remarkable improvements in both adsorption capacities and kinetics compared to GO alone, with removal efficiencies often exceeding 95–99% within minutes.^68,72^ Magnetic modification further enables rapid separation and reuse, one of the most critical advantages of real-world deployment, as many MGOs maintain performance over five or more regeneration cycles.^45,62^ More specifically, GFeN and Fe_3_O_4_–GO hybrids demonstrated exceptionally high adsorption capacities for both As(iii) and As(v), exceeding even 300–400 mg g^−1^ and showed the synergistic role of GO sheets in dispersing iron nanoparticles and preventing aggregation.^72^ More advanced composite architectures have been developed to further enhance selectivity, stability, and scalability. Ternary systems such as GO/CNT/Fe_3_O_4_ showed improvement in conductivity, structural stability, and resistance against competing ions, which could achieve almost complete removal of arsenic (≈99%) with high Langmuir correlation.^63^ Similarly, biopolymer-assisted composites like GO–goethite–chitosan and GO–FeO_*x*_–chitosan beads resulted in enhanced immobilization, bead recoverability, and column applicability along with high adsorption capacities (64. 70). Ion-imprinted MGO systems further improved selectivity toward As(iii), retaining up to 75% adsorption capacity after multiple reuse cycles.^69^ Hybrid materials integrating metal–organic frameworks, hydrogels, or zeolites with GO have expanded the functional scope of arsenic adsorbents. UiO-66–GO composites combined high surface area with structural robustness while showing strong reusability through acid regeneration,^67^ and 3D magnetic GO hydrogels allowed ultra-fast adsorption due to macroporous architectures and preserved oxygen functionalities.^74^ Zeolite–GO and clinoptilolite-based composites enhance ion-exchange and electrostatic interactions, demonstrating promising column-scale performance in simulated waters.^71^ Additionally, rGO–TiO_2_ and Fe_2_O_3_/TiO_2_ hybrids introduced photocatalytic oxidation pathways, accelerating As(iii) to As(v) conversion and improving overall removal efficiency under light-assisted conditions.^73,75^ Overall, comparative literature evidence shows that GO-iron and magnetic nanohybrids exhibit better arsenic removal performance, reusability, and operational flexibility than conventional adsorbents. Recent pilot-scale studies and lifecycle assessments have reiterated their cost-effectiveness and scalability, hence positioning the MGO-based systems as one of the most promising candidates for use in groundwater and wastewater treatment sustainability applications.^62,65^Table 1 goes into additional detail about the many types of GO and its derivatives, their synthesis methods, particular uses for water treatment, and associated experimental procedures and outcomes. In conclusion, the widespread occurrence of As at hazardous amounts and the shown efficacy of GO-based nanoparticles underscore the pressing need to create and implement such technologies to safeguard water quality globally.

## 6. Factors affecting arsenic removal efficiency of graphene-based materials

The removal efficiency of As by graphene-based materials from water and soil is influenced by multiple factors, including functionalization and modification, dimensionality and porosity, pH of the aqueous solution, coexisting ions, and regeneration and reusability.

### 6.1 Functionalization and modification

The surface chemistry of pristine graphene limits its interaction with As due to its property of hydrophobicity. Surface functionalization of pristine graphene introduces many functional groups onto the surface of graphene, such as carboxyl group (–COOH), hydroxyl group (–OH) and epoxy group (–O–). This form of graphene is called graphene oxide, which is hydrophilic in nature and suitable for attracting As ions present in aqueous environments. The oxygen functional groups present on the surface of graphene enhance the adsorption capacity of the material by facilitating electrostatic interaction and surface complexation with arsenic species.^77^ These functional groups play a key role in the modification of the surface chemistry of graphene. These functional groups having negative charges on them attract positively charged As(iii) species towards them at near neutral pH, facilitated by hydrogen bonding. The type and density of functional groups present on the surface of graphene oxide can be precisely controlled, which allows graphene to have adsorption properties for specific arsenic species in the wastewater. For example, amine groups on the surface of graphene oxide target As(v) specifically through electrostatic interaction.^78^

Graphene oxide along with metal oxide and metal nanoparticles has been proven to be an efficient material for removing arsenic from water and soil. Several studies reported that graphene oxide, along with other nanomaterials combines to form graphene-based nanocomposites that have removal rates of arsenic more than 99%. These nanocomposites include magnetic graphene oxide (MGO), graphene oxide iron nanohybrid (GFeN), zerovalent iron nanoparticles and graphene oxide hybrid (*n*ZVI/GO) and iron oxide–graphene oxide (FeO_*x*_–GO) nanocomposites, *etc.*.^76,79^ Along with oxygen functional groups present on the surface of graphene oxide, metal oxides provide additional binding sites for arsenic species to increase overall adsorption capacity. Metal oxides also oxidize As(iii) into a less toxic form As(v), a form of arsenic that can be easily absorbed by graphene-based materials.^80^ The synergistic effect between graphene oxide and metal oxides tremendously improves the overall adsorption capacity of the material. The choice of metal oxide and the method of its incorporation greatly influence the adsorption capacity of the overall material^81^

### 6.2 Dimensionality and porosity of graphene-based nanomaterials

Graphene-based material having a two-dimensional (2D) structure tends to aggregate overtime, which reduces the overall surface area available for arsenic species. On the other hand, graphene-based materials with three-dimensional (3D) structures overcome the limitation of aggregation by having interconnected porous structures such as aerogels and hydrogels.^77^ Three-dimensional graphene composites like Mg–Al LDH/GO_2_ demonstrated 183.11 mg g^−1^ adsorption of As(v) when made from functional graphene sheets.^82^ Porous graphene oxide-nickel ferrite nanocomposites demonstrate exceptional efficiency, achieving >99.9% arsenic removal due to increased pore numbers and adsorption sites that reduce nanoparticle aggregation while maintaining a high surface area. The enhanced performance is attributed to graphene's unique physicochemical properties, including high surface area, porosity, and mechanical flexibility.^4^ Hence, 3D graphene-based materials have more surface area and stability in complex water systems as compared to 2D graphene-based structures.^5,6^ The synthesis method of these graphene-based materials directly influences the porosity of the material. For example, a coprecipitation method was used to synthesize a nanocomposite of graphene oxide with nickel and iron salt, resulting in a highly porous material with enhanced adsorption capacity.^7^

### 6.3 pH of the aqueous solution

pH variation significantly affects As removal utilizing the graphene-based nanomaterials through multiple mechanisms. Graphene oxide-supported nanoscale zero-valent iron (GNZVI) demonstrates high removal efficiency, achieving >90% removal efficiency for both As(III) and As(v) across a wide pH range,^3–9^ while magnetite-graphene oxide composites (GM) show high efficiency (>90%) only at pH 3.^8^ The pH-dependent corrosion products of iron-based graphene composites also influence adsorption affinity, with higher pH favouring magnetite formation that enhances As(v) removal.^9^ Functional graphene sheets achieve a maximum As(iii) adsorption capacity of 138.79 mg g^−1^, while Mg–Al LDH/GO_2_ shows the highest As(v) capacity of 183.11 mg g^−1^.^82^ The presence of an oxygen functional group on the surface of graphene oxide makes the structure negative in nature. Graphene oxide at a near-neutral pH value tends to attract more As(iii) species efficiently, which results in increased adsorption capacity. At the lower pH values the surface of graphene oxide becomes less negative and hence decreases the adsorption capacity.^77^ The removal mechanisms differ by As(v) removal, which is primarily controlled by electrostatic attraction between positively charged adsorbent surfaces and anionic As(v) species, while As(iii) removal occurs through surface complexation and ligand exchange rather than electrostatic interactions.^83,84^

### 6.4 Coexisting ions

Graphene-based materials for arsenic removal are critically influenced by competing ions in wastewater treatment. Graphene-based adsorbents typically contain high concentrations of coexisting anions, such as phosphate (PO_4_^3−^), sulphate (SO_4_^2−^), and carbonate (CO_3_^2−^) that compete with arsenic for adsorption sites on graphene-based adsorbents. Due to its structural similarity and coordination behavior, phosphate is often highly competitive with arsenate/arsenite for the same binding sites, resulting in dramatic reductions in arsenic adsorption efficiency. The removal efficiency of sulphate and carbonate may be less under certain conditions.^63,85^ For adsorbents to remain effective under realistic, multi-ion water conditions, functionalization or composite formation must be used to maintain high selectivity. For graphene-based remediation technologies to be applied effectively, it is essential to evaluate arsenic removal under these competitive conditions.

In treatment scenarios involving real water matrices, the simultaneous presence of various ions greatly influences the adsorption behavior of arsenic on graphene-based adsorbents. Besides arsenite and arsenate, competitive solutes infiltrate of the finite binding sites on the graphene surface. The intensity of this competition is governed by solute loadings, the chemical identity of the ions, their respective binding affinities, the nature and distribution of active centers on the adsorbent. Phosphate, sulfate, and bicarbonate, for instance, adsorb co-planarly with arsenic and drive down the arsenic uptake, a phenomenon quantified in recent reports.^78,80^ Concurrent cationic species, notably calcium and magnesium, modulate the surface charge density of the graphene composites, with consequent shifts in the effective arsenic load. Before moving to large-scale applications, it is necessary to thoroughly study how competing ions affect arsenic removal due to the interaction between dissolved substances and arsenic in various types of water. It has also been proven that phosphate significantly reduces arsenic adsorption as well. Electrospinning iron-functionalized chitosan nanofibers showed significantly reduced arsenic uptake when the water contained high levels of phosphate.

### 6.5 Regeneration and reusability

For environmental sustainability and economic viability, regeneration and reusability of graphene-based materials are crucial. The optimum regeneration process depends on the type of material being used and the adsorption mechanism.Regeneration and reusability are both critical in making graphene-based absorbents for arsenic removal practical and cost-effective. Acid/base washes or salt solutions, thermal treatment, and rinsing with solvents have been reported in literature as common chemical desorption techniques; magnetically separable graphene composites make recovery and repeated use easy.^67^ Several graphene-based composites demonstrate good short-term reuse without obvious capacity decline, but they gradually lose capacity over cycles. Magnetite-graphene and other metal-oxide/graphene hybrids are most frequently emphasized to have ease of separation and multiple reuse cycles with acceptable performance, which makes them attractive for scale-up. Biopolymer-graphene composites, like chitosan-iron-GO beads, allow for effective desorption and reuse while offering improved mechanical stability and handling.^70^ However, these repeated regenerations may lead to changes in the structure or surface chemistry, metal leaching in the case of loaded composites, and a decline in capacity. Thus, adsorption–desorption tests under realistic water chemistries and long-term leaching/stability assessments must be performed before field deployment.

On the other hand, the life cycle of graphene-based materials is an important factor in terms of environmental sustainability. Some graphene-based materials have shown excellent reusability over multiple cycles, maintaining high adsorption capacity. For example, studies have shown that magnetic graphene oxide (MGO) and graphene oxide iron nanohybrid (GFeN) are reusable of materials over multiple cycles with great efficiency.^79,83^ On the other hand, some graphene-based materials showed loss in adsorption capacity after some cycles.^86^

## 7. Removal mechanism of As from wastewater using graphene-based nanomaterials

**Fig. 4:**
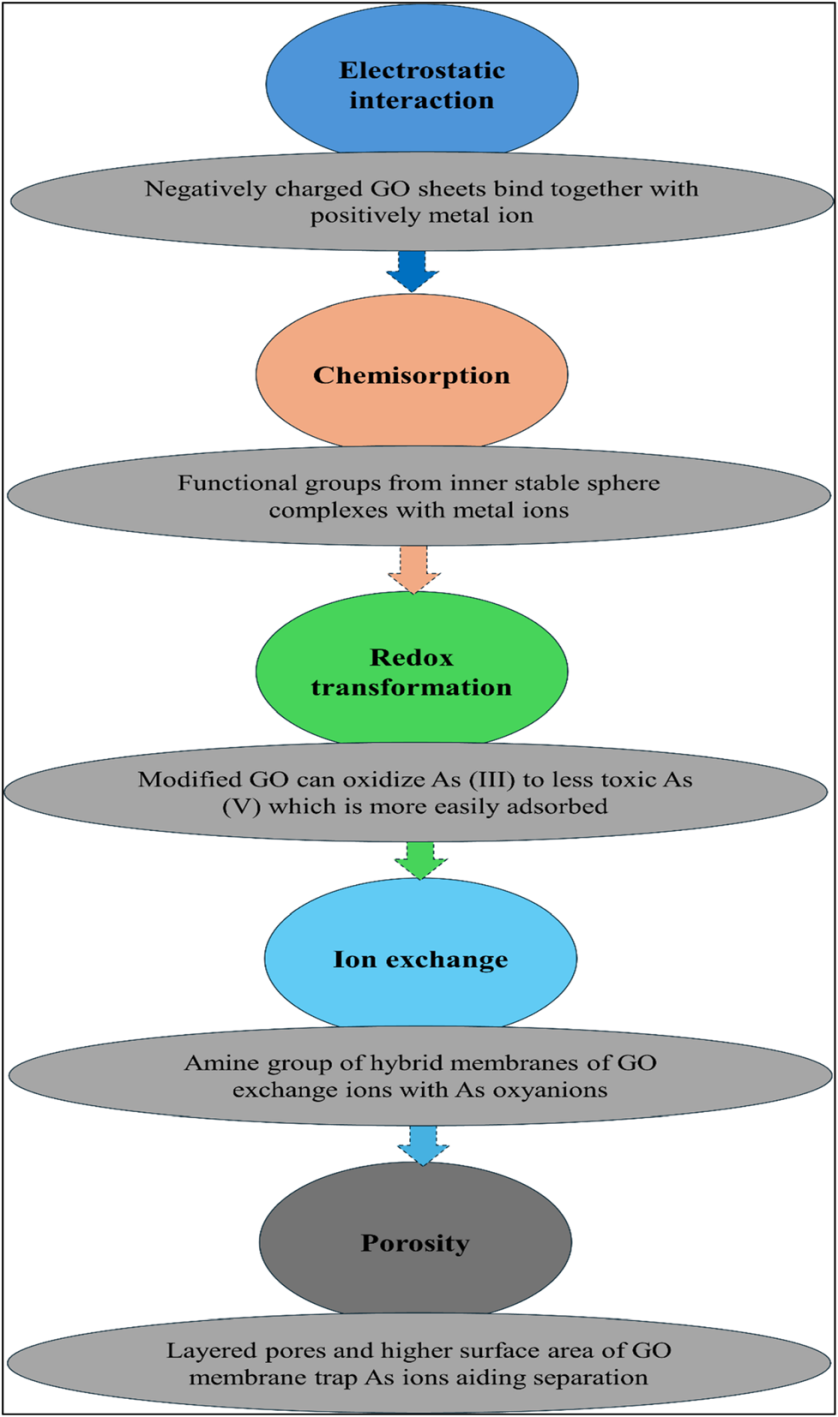
Mechanism of HMs removal especially the arsenic from wastewater utilizing the graphene-based nanomaterials.

In order to remove arsenic easily from graphene-based nanomaterials, notably graphene oxide (GO), reduced GO, and GO-based hybrids, a synergistic process is used (i) at metal-oxide sites attached to graphetic sheets through inner-sphere surface complexation (ii) by oxygenated or amine-bearing groups through electrostatic attraction, and (iii) by *in situ* redox conversion that oxidizes As(iii) into As(v), which is more readily adsorbable. Graphene oxide decorated with iron oxide forms stable Fe–O–As inner-sphere complexes by substituting surface hydroxyls with arsenate/arsenate, resulting in high capacities and fast kinetics of GO–Fe nanohybrids.^71,83^ As a complement to chemisorption, electrostatic/ion-exchange interactions with GO –COO/–OH groups and protonated amines in biopolymer hybrids (*e.g.*, chitosan) increase arsenate oxyanions while improving membrane integrity and flux. Arsenic speciation studies indicated that As(v) is primarily predominant as CaAsO^4^ (47%) and MgHAsO_4_^0^ (32%) in seawater, whereas the predominant As chemical species in fresh water are HAsO_4_^2^− (31%) and H_2_AsO_4_ (31%) along with CaHAsO_4_^0^ (26%).^87^ Similarly, the most prevalent form of As is As(iii) under a reduced environment (low pH value)^88^ and oxoanion speciation depends on pH *i.e.*, arsenous acid H_3_AsO_3_ (up to pH ≈ 9) and the stable anionic species H_2_AsO_3_ (pH ≈ 9–11). Chelation is also occurring as a result of interaction between arsenic and chelating agents that ultimately affect metal availability.^87,89^ During removal/immobilization of other cationic metals, the anionic nature of As species results in low efficacy of remediation. Additionally, Fe(ii) incorporation into GO–chitosan enhances As(v) capture by adding additional Fe-site complexation.^90,91^ A graphetic scaffold's high specific area and transport pathways facilitate mass transfer, whereas a customized surface chemistry dictates selectivity. The superior performance and robustness of GO-based arsenic decontamination adsorbents and membranes for arsenic decontamination can be attributed to Fe-rich interfaces, amine-rich matrixes, and ion exchange/electrostatics.^92^ The following Fig. 4 illustrates the arsenic removal mechanism using GO nanomembranes, providing a clear depiction of the As removal process from wastewater.

## 8. Next-generation graphene-based fabricated membranes for As treatment

The fabricated membranes made from graphene have emerged as highly promising materials for the removal of arsenic from soil and water because of their high mechanical strength, separation efficiency, and excellent permeability. Adsorption plays a key role in capturing As species from contaminated environments through these membranes. Among the different types, graphene oxide–polymer hybrid membranes are recognized for their strong mechanical properties and efficient As removal, where the functional groups of GO adsorb As ions, while the polymer matrix enhances stability and filtration performance with electrostatic interactions. The hybrid graphene oxide–chitosan membranes use GO for adsorption and chitosan's amino groups for ion exchange, thereby eliminating As(iii) and As(v).^93^ Through chemical interactions between arsenic ions and ZnO NPs embedded within GO, GO–ZnO nanocomposite membranes demonstrate high As adsorption capacity, providing a large surface area and stable surface complexes that are efficient in capturing arsenic. Furthermore, graphene oxide–cellulose membranes have a strong affinity for As(v), since GO facilitates electrostatic adsorption and cellulose supports filtration.^94^ By forming strong inner-sphere complexes between Fe and As species, functionalized GO membranes with iron oxide further improve As uptake of both As(iii) and As(v). By maximizing surface area and offering multiple adsorption sites through a combination of physical and chemical processes, nanohybrid GO–activated carbon membranes enhance removal efficiency.^95^ Due to their large surface areas and chemical adsorption properties, reduced graphene oxygen (rGO) nanocomposites demonstrate excellent binding capacity for As species, particularly when combined with metallic nanoparticles. A second efficient route is to modify GO with iron hydroxide, where ligand exchange produces Fe–As complexes, enabling As(v) to be removed more efficiently. In addition, nitrogen-doped graphene membranes enhance adsorption by introducing electron-rich sites that strengthen electrostatic interactions with As(v) ions.^96^ Their oxygen-rich functional groups allow them to promote ion exchange and electrostatic adsorption, environmentally safe nano green functionalized GO membranes, which are made with green chemistry techniques, offer long-term solutions for the removal of arsenic.^97^ The following Table 2 presents an overview of several high-performance graphene-based membranes, highlighting their synthesis pathways and key functional properties by summarizing these next-generation membranes and their broad applications in various aspects of daily life.

**Table 2:** Next-generation production of high-quality graphene membranes through advanced synthesis techniques for cutting-edge applications of graphene nanosheets

Graphene membranes	Method of preparation	Salient properties	References
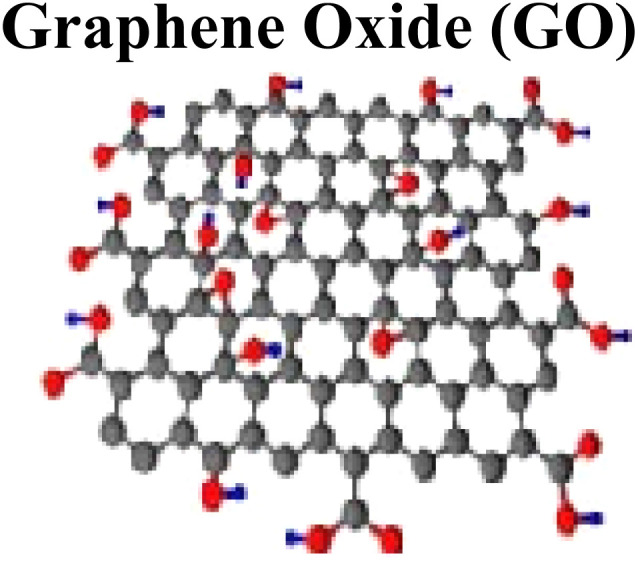	Vacuum filtration, layer-by-layer assembly of GO nanosheets	Hydrophilic, oxygen functional groups, tunable pores	98
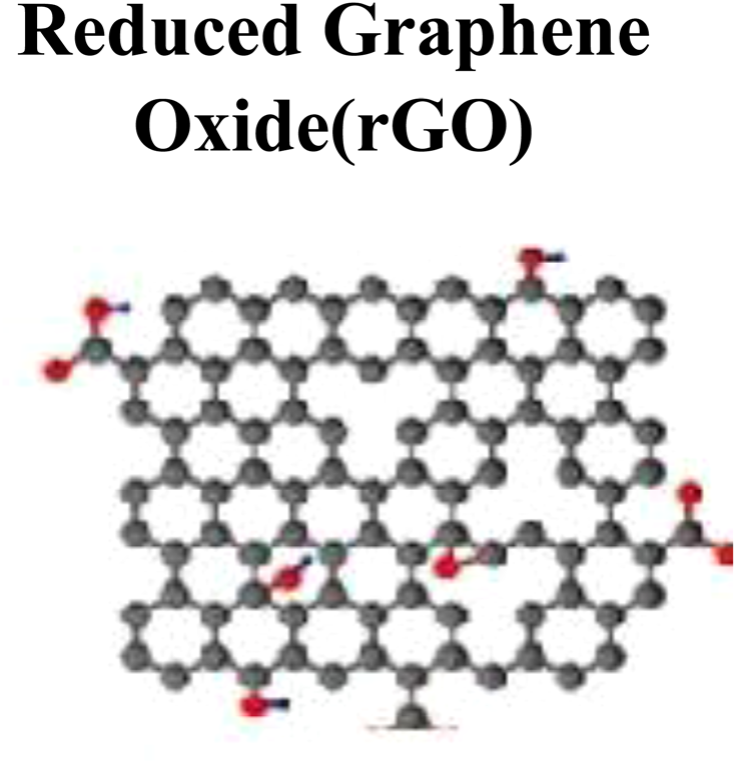	Chemical, thermal, or electrochemical reduction of GO	Hydrophobic, conductive, improved stability	98
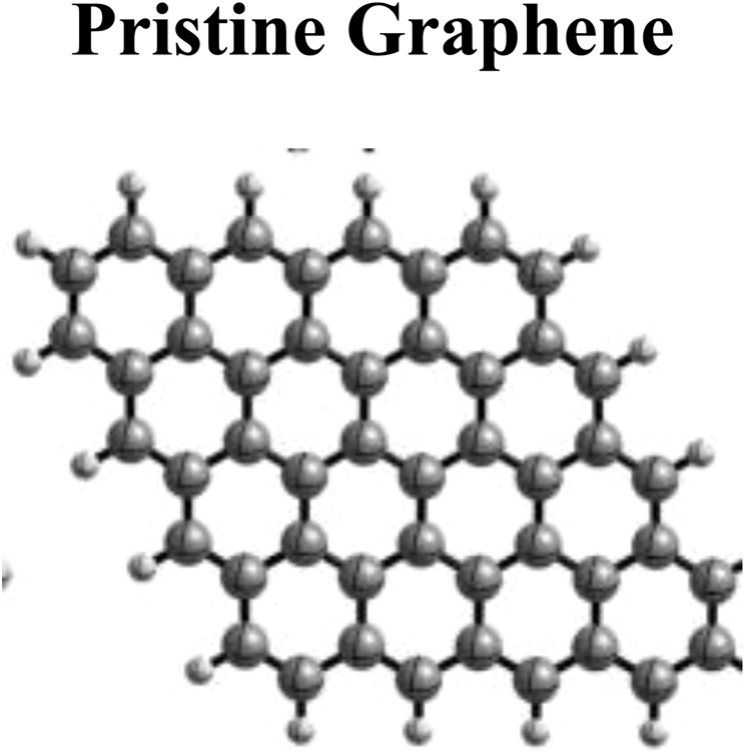	Defect engineering, nanoporous graphene sheets	Ultrahigh permeability, molecular sieving	99
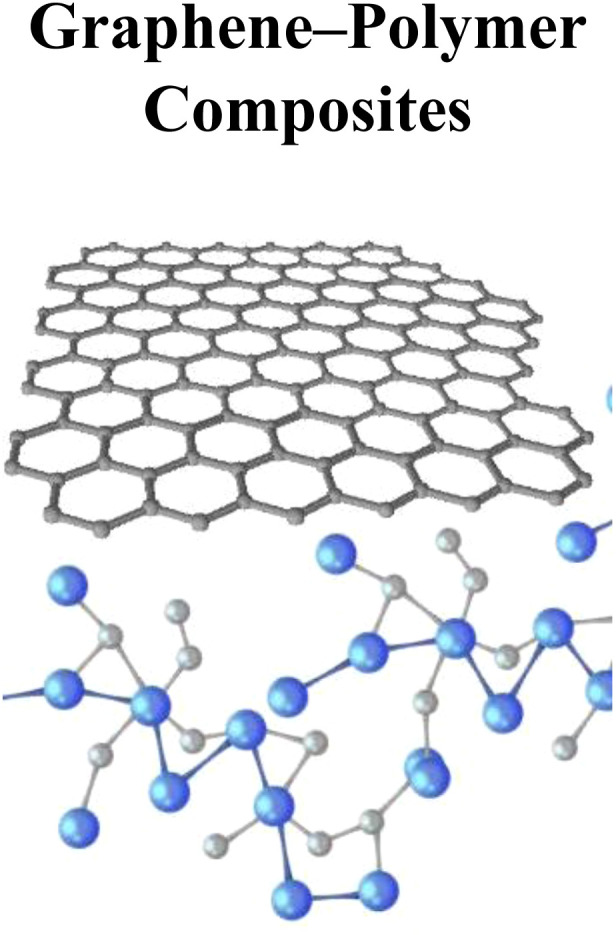	Blending GO/rGO with polymers (PSf, PVDF, polyamide)	Strong, anti-fouling, selective rejection	100
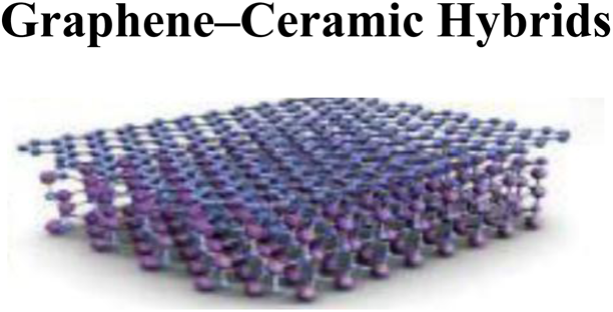	Embedding graphene with ceramic supports (alumina, silica)	High thermal/chemical stability, reusable	101
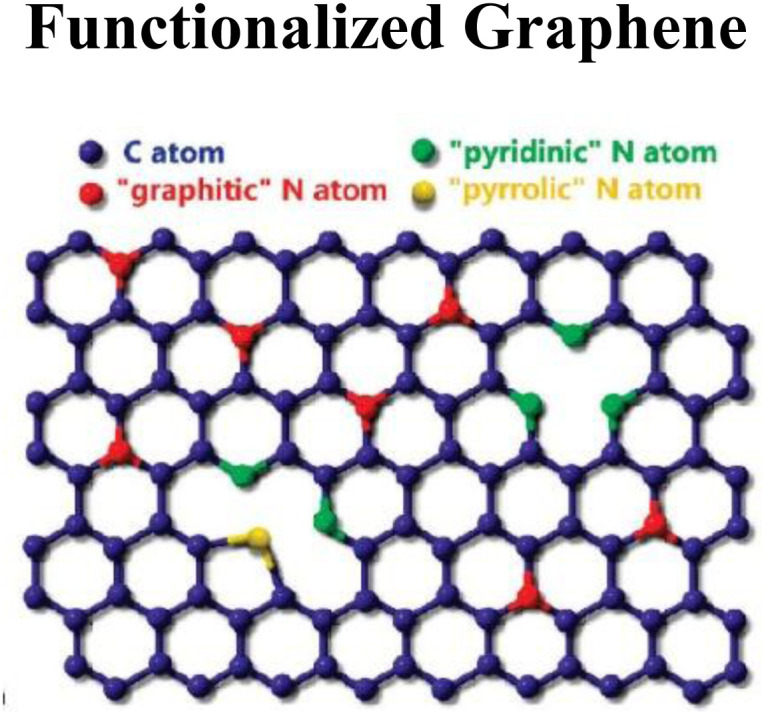	Surface modification with nanoparticles (Ag, TiO_2_, Fe_3_O_4_) or groups	Antibacterial, photocatalytic, enhanced adsorption	102
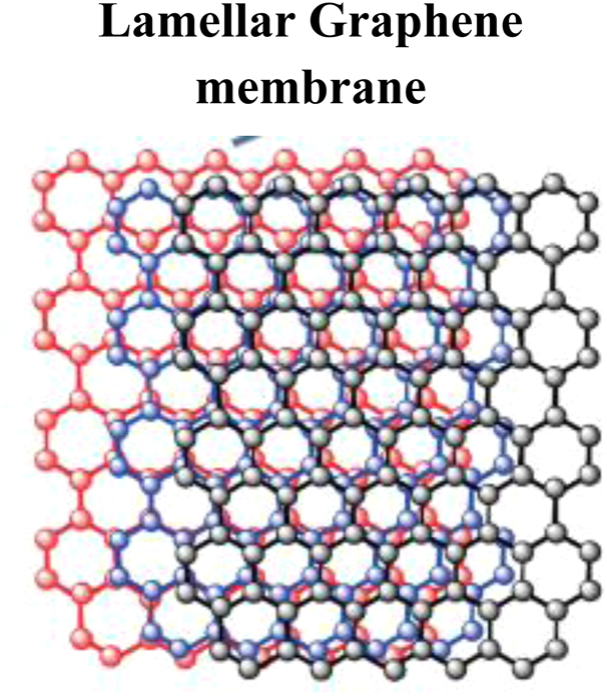	Self-assembly into hydrogels/aerogels	Porous, high flux, strong trapping ability	103
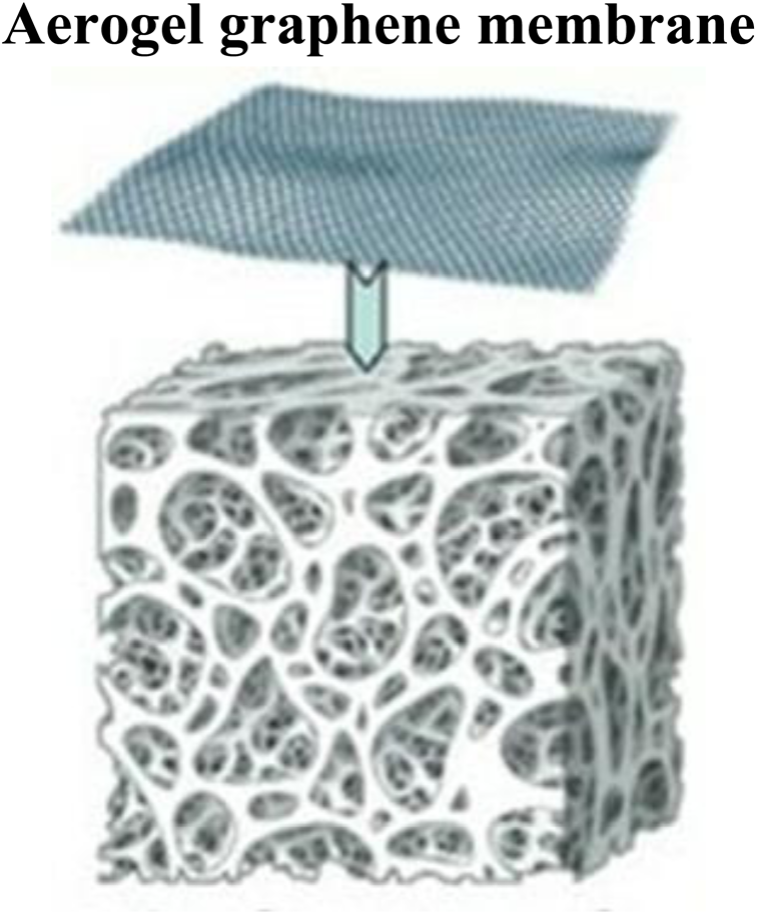	Combination of aerogel and graphene oxide	Flexible, high porous in nature, sticky surface	104

**Fig. 5:**
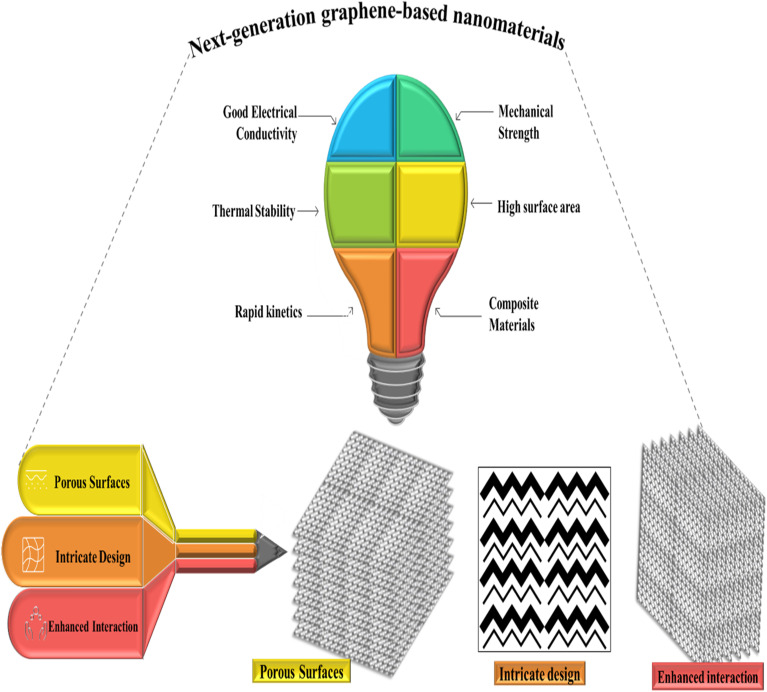
Design, properties, types and important characteristics of next-generation graphene-based nanomaterials.

Graphene oxide (GO)-based nanomembranes have been developed and applied for the separation and purification of HMs from wastewater, as shown in Table 2. For the development of next-generation GO-based membranes by forming multilayer and ultrathin structures with the interconnected chain-like architecture of GO, highly porous membranes with enhanced separation efficiency can be developed. The structural advantages of nanomembranes that are tailored and applied to specific applications help them achieve these properties (Fig. 5). To promote efficient binding and removal of As ions and suspended contaminants, nanomaterials should be engineered with interconnected porous networks, intricate architectures, and enhanced surface interactions. To ensure durability and superior performance, next-generation nanomembranes must exhibit high mechanical strength, large surface areas, composite compatibility, thermal stability, good thermal conductivity, and rapid reaction kinetics.

## 9. Conclusion

The severe impacts of arsenic contamination on human health and ecosystems make it a pressing global challenge. Although several technologies have been developed for the remediation of arsenic, many of these technologies remain difficult to access, expensive, or complex for communities most affected by contamination. Among emerging solutions, graphene-based nanomaterials, particularly graphene oxide (GO), reduced graphene oxide (rGO), and their functionalized hybrid membranes, have demonstrated remarkable potential due to their large surface area, tunable functional groups, strong adsorption capacity, and reusability. The present review provides a deeper insight into the emerging generation of graphene-based nanomembranes designed for more efficient removal of arsenic (As) and other contaminants from aqueous solutions. As a result of the abundance of oxygen-containing functional groups, graphene nanomaterials possess exceptional adsorption abilities, creating multiple active binding sites for removing metal and metalloid ions from wastewater by creating multiple active binding sites. In addition, eco-friendly alternatives such as green-synthesized, functionalized GO membranes offer promising and sustainable pathways for future large-scale applications. The improved surface chemistry, synergistic functionality, and superior interaction with arsenic species of a variety of graphene-based composites, including GO-polymer hybrids, GO-chitosan, GO-ZnO, and iron-functionalized GO, enhance adsorption efficiency for both As(iii) and As(v) by significantly enhancing adsorption efficiency. Therefore, future studies must concentrate on creating scalable, affordable, and environmentally safe nano green technologies that are verified in real-world settings. By overcoming these obstacles, graphene-based adsorption technologies may offer a viable and sustainable solution to reduce As exposure and protect water supplies for populations that are at risk globally.

## Author contributions

M. S. A.; conceptualization, supervision, resources, writing – review & editing. M. A. I.: conceptualization, writing – original draft, writing – review & editing, A. H.: writing – original draft, writing – review & editing. M. I.: writing – original draft, visualization; L. I.; data curation, writing – review & editing; M. A. R.; resources, writing – review & editing; Y. N.: conceptualization, writing – original draft.;. S. A. A.; visualization, data curation. Writing – original draft, A. I.: formal analysis (literature survey), visualization, funding acquisition, writing – review & editing. M. E. A. Z.: funding acquisition, project administration, formal analysis (literature survey), writing – review & editing.

## Conflicts of interest

The authors declare that they have no known competing financial interests or personal relationships that could have appeared to influence the work reported in this paper.

## Funding

This work was supported and funded by the Deanship of Scientific Research at Imam Mohammad Ibn Saud Islamic University (IMSIU) (grant number IMSIU-DDRSP2602).

## Data Availability

As this is a review article, no new experimental data, code, or software were generated or analyzed. All data supporting the conclusions of this review are included within the article and its referenced sources.
